# Echocardiographic features of right ventricle in septic patients with elevated central venous pressure

**DOI:** 10.1186/s12871-024-02515-8

**Published:** 2024-04-04

**Authors:** Hongmin Zhang, Dingding Zhang, Hui Lian, Qing Zhang, Xiukai Chen, Xiaoting Wang

**Affiliations:** 1 Department of Critical Care Medicine, Peking Union Medical College Hospital, Chinese Academy of Medical Sciences and Peking Union Medical College, 1# Shuai Fu Yuan, Dong Cheng District, Beijing, 100730 China; 2 Critical Care Ultrasound Study Group, Beijing, China; 3 Medical Research Center, State Key Laboratory of Complex Severe and Rare Diseases, Peking Union Medical College Hospital, Chinese Academy of Medical Sciences and Peking Union Medical College, Beijing, China; 4 Department of Health Care, Peking Union Medical College Hospital, Chinese Academy of Medical Sciences and Peking Union Medical College, Beijing, China; 5 Department of Cardiopulmonary Science, Respiratory Care Division, Rush University, Chicago, IL USA

**Keywords:** Sepsis, Echocardiography, Central venous pressure, Right ventricular failure, Prognosis

## Abstract

**Background:**

Elevated central venous pressure (CVP) is deemed as a sign of right ventricular (RV) dysfunction. We aimed to characterize the echocardiographic features of RV in septic patients with elevated CVP, and quantify associations between RV function parameters and 30-day mortality.

**Methods:**

We retrospectively reviewed a cohort of septic patients with CVP ≥ 8 mmHg in a tertiary hospital intensive care unit. General characteristics and echocardiographic parameters including tricuspid annular plane systolic excursion (TAPSE), pulmonary vascular resistance (PVR) as well as prognostic data were collected. Associations between RV function parameters and 30-day mortality were assessed using Cox regression models.

**Results:**

Echocardiography was performed in 244 septic patients with CVP ≥ 8 mmHg. Echocardiographic findings revealed that various types of abnormal RV function can occur individually or collectively. Prevalence of RV systolic dysfunction was 46%, prevalence of RV enlargement was 34%, and prevalence of PVR increase was 14%. In addition, we collected haemodynamic consequences and found that prevalence of systemic venous congestion was 16%, prevalence of RV-pulmonary artery decoupling was 34%, and prevalence of low cardiac index (CI) was 23%. The 30-day mortality of the enrolled population was 24.2%. In a Cox regression analysis, TAPSE (HR:0.542, 95% CI:0.302–0.972,* p* = 0.040) and PVR (HR:1.384, 95% CI:1.007–1.903,* p* = 0.045) were independently associated with 30-day mortality.

**Conclusions:**

Echocardiographic findings demonstrated a high prevalence of RV-related abnormalities (RV enlargement, RV systolic dysfunction and PVR increase) in septic patients with elevated CVP. Among those echocardiographic parameters, TAPSE and PVR were independently associated with 30-day mortality in these patients.

**Supplementary Information:**

The online version contains supplementary material available at 10.1186/s12871-024-02515-8.

## Background

The right ventricle (RV) has been the focus of renewed interest in recent years [1–4]. RV affects venous return primarily by lowering central venous pressure (CVP). Thus, CVP elevation can be seen as a sign that venous return has exceeded the limit of RV accommodation and is an important marker of RV failure [5]. A prior study argued that, CVP ≥ 8 mmHg in combination with RV enlargement could be a sign of RV failure in septic shock patients [6].

Echocardiography is the most frequently used imaging modality for RV assessment in the intensive care unit (ICU) due to its ease of use, noninvasive nature, and availability at bedside. Given the complex geometry of the RV, various echocardiographic parameters can be measured including RV systolic function parameters such as tricuspid annular plane systolic excursion (TAPSE), fractional area change (FAC) and tissue Doppler peak velocity of tricuspid annulus (S’), and RV dimension parameters like RV/left ventricular diastolic area ratio(R/LVEDA) [4, 7]. In addition, pulmonary vascular resistance (PVR) can be reliably estimated using echocardiography [8]. We hypothesized that a significant proportion of septic patients with elevated CVP may exhibit RV dysfunction. However, the RV echocardiographic features has not been well recognized in septic patients with elevated CVP. Furthermore, while existing literature has explored the prognostic implications of RV systolic function and RV dilation in sepsis, these investigations have not accounted for PVR [9, 10]. The aim of our research is to delineate the echocardiographic features of the RV and correlate these measures with mortality at a 30-day interval in septic patients with a CVP of 8 mmHg or higher.

## Patients and methods

### Study population

This is a retrospective study on septic patients admitted from June 2018 through February 2023 at a tertiary hospital ICU. We screened septic patients with transthoracic echocardiography (TTE) examination for enrolment.

Sepsis and septic shock were characterized by the Sepsis-3 definition [11]. Sepsis was defined as a life-threatening organ dysfunction caused by a dysregulation of the host response to infection. Alternatively, septic shock was characterized if sepsis patients displayed a persisting hypotension requiring vasopressors to maintain a mean arterial pressure (MAP) ≥ 65 mmHg, and the association of a serum lactate level > 2 mmol/L after adequate fluid resuscitation.

Patients were excluded if they withheld life support or had a history of chronic heart failure or cardiac surgery, acute coronary syndrome within 1 week, left ventricular outflow tract (LVOT) obstruction [12], prosthetic valves or severe valvular diseases, moderate to severe chronic pulmonary hypertension, diagnosis of elevated intraabdominal hypertension [13], or inadequate echocardiographic images for measurement. Patients also would not be enrolled if they lack CVP monitoring or had a CVP below 8 mmHg.

The study was conducted in compliance with the Declaration of Helsinki and was approved by the ethics committee of our institution (Approval No. I-23PJ1278). Informed consent was waived due to the retrospective nature of this study.

### Echocardiography

Ecocardiography was routinely performed in this critical care setting for sepsis patients within 24 h of admission. TTE (Mindray, M9, Shenzhen, China) was performed by physicians with advanced training, with parasternal longitudinal- and short-axis views, apical four- and three-chamber views, and subcostal four-chamber and inferior vena cava longitudinal views being obtained for measurements. Images were saved for offline analysis. At least three cardiac cycles were analysed and averaged. The echo results were interpreted based on the PRICES statement [14]. Intraobserver and interobserver variability for key measures of cardiac function by the performers have been previously published [15].

RV-related abnormalities were assessed regarding RV dimension, RV systolic function and PVR. RV enlargement was represented by R/LVEDA from apical 4-chamber view. RV systolic function included TAPSE, FAC, and S’. The measurements of TAPSE, FAC and S’ were obtained as previously described [15, 16]. PVR was calculated based on the following equation, PVR = TR velocity/VTI_RVOT_ × 10 + 0.16 [17]. Tricuspid regurgitation (TR) was measured from the RV inflow view, apical 4-chamber view and aortic short-axis view via continuous wave Doppler, and the highest value was chosen. Velocity–time integral of right ventricular outflow tract (VTI_RVOT_) was obtained from aortic short-axis view using pulse wave Doppler. R/LVEDA ≥ 0.6, TAPSE < 1.7 cm, S’ < 9.5 cm/s, FAC < 35%, and PVR > 3 wood units were considered abnormal [6, 18–20].

Haemodynamic consequences included systemic venous congestion, RV-PA decoupling and low cardiac output. The inferior vena cava internal diameter (IVCD) was measured in the subcostal longitudinal plane, just upstream of the origin of the suprahepatic vein at end expiration. The HV Doppler was also obtained from the IVC subcostal longitudinal plane using pulsed wave Doppler at the end of respiratory phase. Systemic venous congestion was decided based on IVCD and hepatic vein Doppler: IVCD > 20 mm and hepatic vein S < D [21]. RV-pulmonary artery (PA) coupling was estimated with TAPSE/pulmonary arterial systolic pressure (PASP), TAPSE/PASP ≤ 0.5 mm/mmHg was considered as RV-PA decoupling [16]. The PASP was calculated by the following equation: PASP = 4 × (TR velocity)^2^ + CVP. CI < 2.5 L/min/m^2^ was considered abnormal [22]. LVOT-VTI was measured using pulsed wave Doppler from apical 5-chamber view or 3-chamber view. Stroke volume (SV) was calculated as π × (LVOT diameter/2)^2^ × LVOT-VTI and CI was calculated as strove volume index × heart rate (HR).

### Clinical data collected

We collected the patients’ demographic information, Acute Physiology and Chronic Health Evaluation (APACHE) II score, and Sequential Organ Failure Assessment (SOFA) score at ICU admission. Each patient’s HR, MAP, CVP, norepinephrine (NE) dose, positive end-expiratory pressure (PEEP), plateau pressure (Pplat), and volume of fluid expansion were also collected at the time of the echo examination. CVP monitoring was a common procedure in septic patients and was measured through a central venous catheter by placing the transducer at mid-axillary level fourth intercostal space while the patient was lying supine [23].

### Outcomes

The primary endpoint of the study was a 30-day survival rate and the secondary endpoints were the haemodynamic alterations including systemic venous congestion, RV-PA decoupling and low CI in those patients.

### Statistical analysis

Continuous variables are expressed as the median and interquartile range according to their distribution. Categorical variables are presented as frequencies and percentages. Prognostic factors for a 30-day mortality were determined using univariate and multivariate Cox regression models. Based on the results of the univariate Cox regression model, the baseline covariables with *p* values < 0.1 were selected by the stepwise method, age, SOFA score, APACHE II score, PEEP, Pplat, norepinephrine dose, and whether the patient was diagnosed with lung infection were included in the multivariate Cox regression models as covariables and the hazard ratio was calculated together with its 95% confidence intervals. Statistical analyses were conducted using SPSS 22.0 and R software 4.2.0. Two-tailed *p* < 0.05 was considered significant.

## Results

### General characteristics

**Fig. 1:**
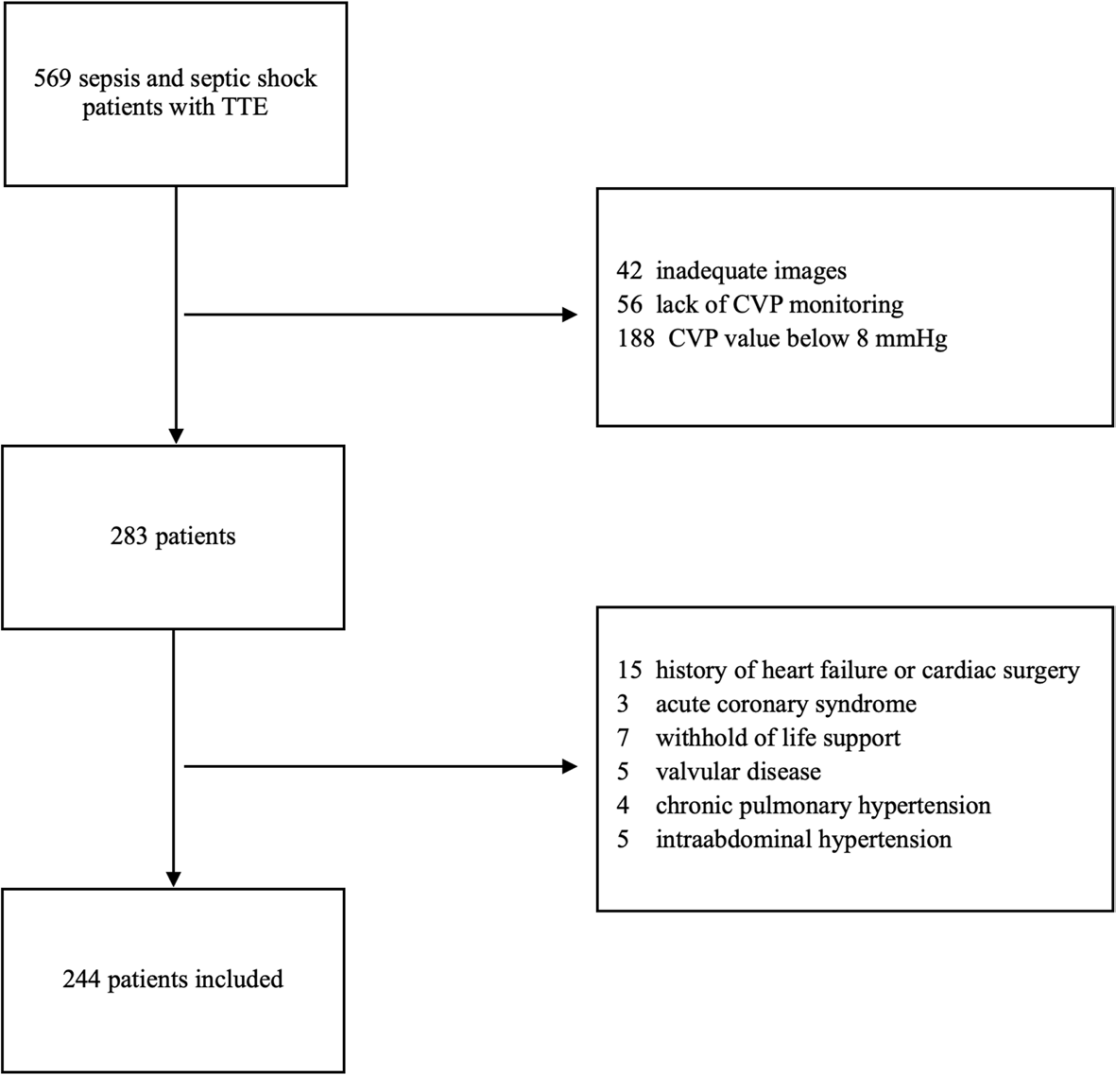
Flow chart of the study. TTE: transthoracic echocardiography; CVP: central venous pressure

A total of 569 septic patients with TTE examination were screened for enrollment. We exlcuded 325 patients, among whom 56 lacked CVP monitoring, and 188 had a CVP value below 8mmHg. A total of 244 septic patients with TTE and a CVP value equal or greater than 8 mmHg were enrolled in this study (Fig. 1). The age was 64 (50, 72) years old. The APACHE II score and SOFA score were 20 (15, 27) and 12 (10, 14), respectively. 85.1% of patients were on NE infusion with a dose of 0.4 (0.2, 0.7) μg/kg/min. The HR and MAP were 97 (83, 108) bpm and 85 (77, 90) mmHg, respectively. The CVP was 9 (9, 11) mmHg. 91% of the patients were on invasive MV support. The length of ICU stay was 6 (3, 11) days (Table 1).

**Table 1:** General characteristics of the patients

Categories	CVP ≥ 8 mmHg (*n* = 244)
Age, years	64 (50, 72)
Male gender(n, %)	154 (63.1)
APACHEII	20 (15, 27)
SOFA	12 (10, 14)
Comorbidities (n, %)	
HTN	114 (46.7)
DM	64 (26.2)
CAD	39 (15.9)
CKD	26 (10.7)
COPD	10 (4.1)
Site of infection (n, %)	
Abdominal	121 (49.6)
Lung	44 (18.0)
Soft tissue	15 (6.1)
Biliary tract	11 (4.5)
UTI	9 (3.7)
^a^Others	44 (18.0)
NE infusion (n, %)	209 (85.7)
NE dose (μg/kg/min)	0.4 (0.2, 0.7)
HR (bpm)	97 (83, 108)
MAP (mmHg)	85 (77, 90)
CVP (mmHg)	9 (9, 11)
MV(n, %)	222 (91.0)
PEEP (cmH_2_O)	7 (5, 8)
Pplat (cmH_2_O)	19 (16, 21)
Fluid expansion (ml)	3667 (2941, 4734)
ICU stay (day)	6 (3, 11)
30-day mortality (n, %)	59 (24.2)

*APACHE* acute physiology and chronic health evaluation, *SOFA* sequential organ failure assessment, *HTN* hypertension, *DM* diabetes mellitus, *CAD* coronary artery disease, *CKD* chronic kidney disease, *COPD* chronic obstructive pulmonary disease, *UTI* urinary tract infection, *NE* norepinephrine, *HR* heart rate, *MAP* mean arterial pressure, *CVP* central venous pressure, *MV* mechanical ventilation, *PEEP* positive end-expiratory pressure, *Pplat* plateau pressure, *ICU* intensive care unit

^a^Others: pleural infection, catheter-related bloodstream infection, intracranial infection, mediastinal infection, and infections of unidentified origin

### Echocardiographic parameters for patients with CVP ≥ 8 mmHg

**Fig. 2:**
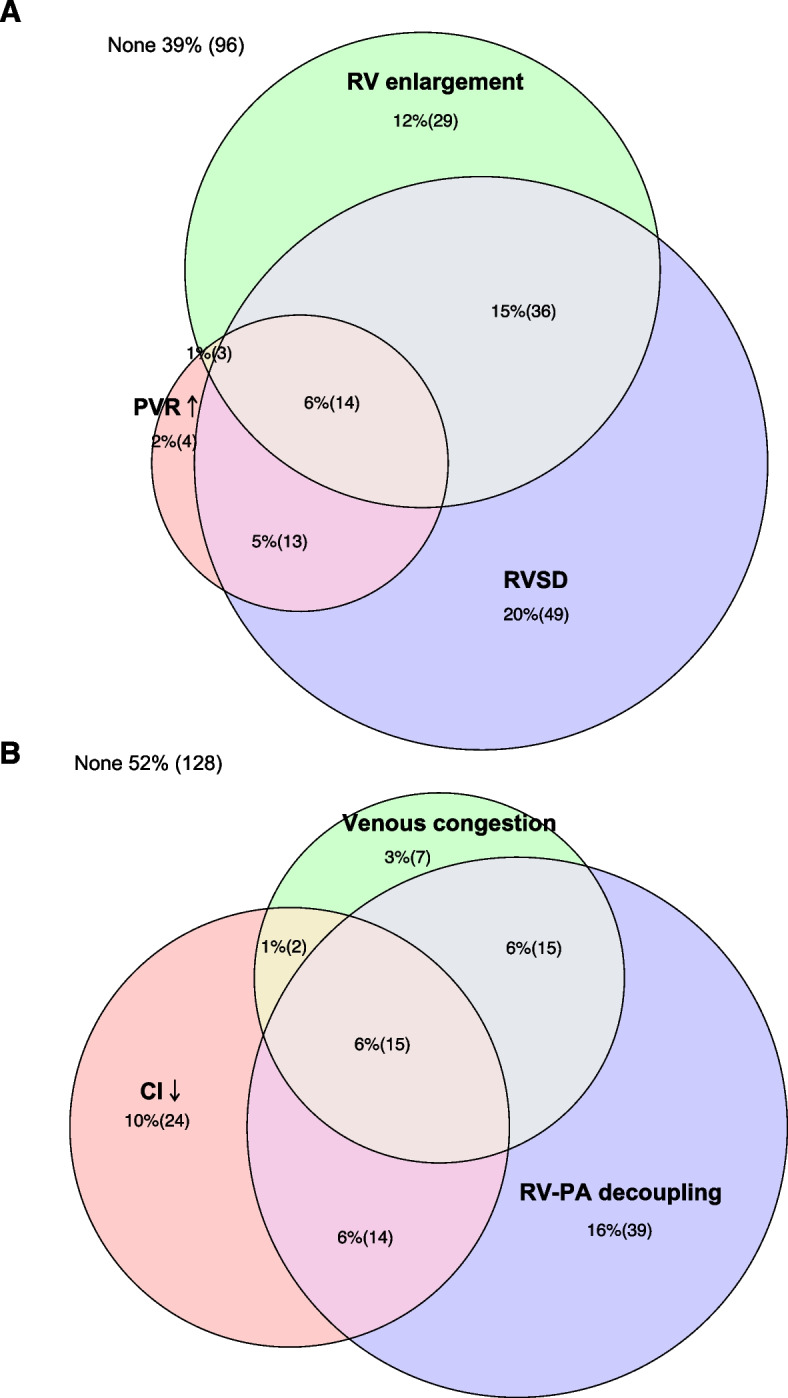
Distribution of haemodynamic and echocardiographic parameters. **A** The distribution of RV-related echocardiographic parameters, demonstrating frequent overlap of RV enlargement, RVSD and PVR increase. **B** The distribution of haemodynamic outcomes, demonstrating frequent overlap of systemic venous congestion, RV-PA decoupling and low cardiac index. TAPSE: tricuspid annular plane systolic excursion; FAC: fractional area change; S’: tissue Doppler peak velocity of tricuspid annulus; RVSD: right ventricular systolic dysfunction; PVR: pulmonary vascular resistance; RV-PA: right ventricle-pulmonary artery; CI: cardiac index

The unavailable measurements were deemed as negative in the proportion calculation. 61% (148/244) of the patients displayed at least one RV abnormalities with RV systolic dysfunction accounting for 46% (112/244), RV enlargement accounting for 34% (82/244), and PVR increase accounting for 14% (34/244). Patients demonstrated appreciable overlap in overall RV appraisal (Table 2, Fig. 2A). TAPSE reduced in 37% (90/244) and FAC reduced in 25% (61/244). 17% (42/244) of the enrolled patients had a S’ < 9.5 cm/s. Left ventricular parameters were listed in Supplemental Table 1.

**Table 2:** Echocardiographic parameters in patients with CVP ≥ 8mmHg (*n* = 244)

Categories	Number of measurement	Findings					Abnormal (n, %)	Definition of abnormal
		Min	25^th^	Median	75^th^	Max		
RV function-related parameters								
R/LVEDA	234	0.24	0.48	0.55	0.65	1.62	82 (34)	≥ 0.6
RV systolic dysfunction								
TAPSE(cm)	242	0.89	1.49	1.90	2.28	3.40	90 (37)	< 1.7
S’(cm/s)	191	2.13	10.3	12.4	14.6	22	42 (17)	< 9.5
FAC(%)	239	10	34	44	52	75	61 (25)	< 35%
PVR (Wood units)	184	1.1	1.8	2.3	2.8	5.7	34 (14)	> 3.0
Haemodynamic consequences								
TAPSE/PASP	216	0.15	0.44	0.59	0.73	1.4	83 (34)	≤ 0.5
Venous congestion	220						39 (16)	IVCD > 2cm and HV S/D < 1
CI(L/min/m^2^)	230	1.1	2.5	3.1	3.8	7.9	55 (23)	< 2.5

*RV* right ventricle, *CVP* central venous pressure, *R/LVEDA* right and left end-diastolic area ratio, *TAPSE* tricuspid annular plane systolic excursion, *FAC* fractional area change, *S’* tissue Doppler peak velocity of tricuspid annulus, *PVR* pulmonary vascular resistance, *PASP* systolic pulmonary arterial pressure, *CI* cardiac index

Patients with haemodynamic consequences accounted for 48% (116/244). In all, TAPSE/PASP was < 0.5 mm/mmHg in 34% (83/244) of patients; systemic venous congestion was confirmed in 16% (39/244) of patients and CI was < 2.5 L/min/m^2^ in 23% (55/244) of patients. Patients demonstrated appreciable overlap in haemodynamic consequences (Table 2, Fig. 2B).

### RV-related echocardiographic parameters and 30-day mortality in patients with CVP ≥ 8 mmHg

**Fig. 3:**
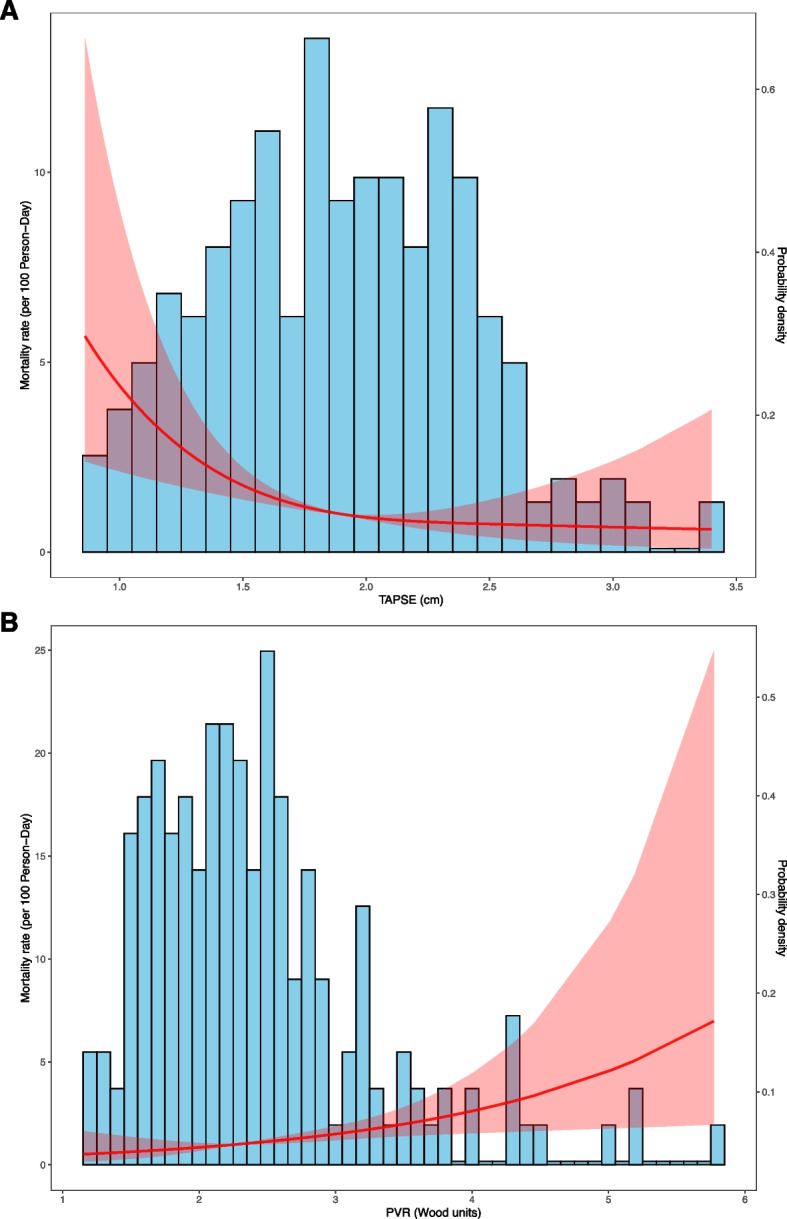
The relationship between echocardiographic measures and 30-day mortality. Continuous association of (**A**) TAPSE (*p* = 0.040) and (**B**) PVR (*p* = 0.045) with mortality was demonstrated. TAPSE: tricuspid annular plane systolic excursion; PVR: pulmonary vascular resistance

At 30 days after ICU admission, 24.2% (59/244) patients died. In the Cox regression analysis, after adjusting for age, APACHE II, SOFA scores, PEEP, Pplat levels, norepinephrine dose, and lung infection, TAPSE (HR:0.542, 95% CI:0.302–0.972,* p* = 0.040) and PVR (HR:1.384, 95% CI:1.007–1.903,* p* = 0.045) were independently associated with 30-day mortality (Fig. 3A, B). R/LVEDA (HR:1.781, 95% CI:0.923–3.439,* p* = 0.085) was also related to 30-day mortality, but was not statistically significant. No association with 30-day mortality was observed with measurements of S’and FAC (Table 3, Supplemental Table 2).

**Table 3:** RV function parameters and 30-day mortality in patients with CVP ≥ 8mmHg

	Dichotomous		Continuous	
	HR (95%CI)	*p*	HR (95%CI)	*p*
R/LVEDA	1.442 (0.812–2.559)	0.212	1.781 (0.923–3.439)	0.085
TAPSE	1.913 (1.058–3.459)	0.032	0.542 (0.302–0.972)	0.040
S’	1.078 (0.514–2.264)	0.842	0.937 (0.858–1.022)	0.141
FAC	1.219 (0.656–2.263)	0.531	0.988 (0.967–1.007)	0.283
PVR	2.260 (1.14–4.626)	0.026	1.384 (1.007–1.903)	0.045

*R/LVEDA* right and left end-diastolic area ratio, *TAPSE* tricuspid annular plane systolic excursion, *S’* tissue Doppler peak velocity of tricuspid annulus, *FAC* fractional area change, *RVOT-FS* right ventricular outflow tract fractional shortening, *PVR* pulmonary vascular resistance

### Sensitivity analysis

We performed sensitivity analysis on patients without lung infection and found that TAPSE (HR:0.399, 95% CI:0.190–0.838,* p* = 0.015) and PVR (HR:1.606, 95% CI:1.033–2.497,* p* = 0.035) were still independently associated with 30-day mortality.

## Discussion

In this study, we investigated the echocardiographic features of the RV in septic patients with CVP ≥ 8 mmHg. We observed a high prevalence of RV-related abnormalities manifested by RV enlargement, RV systolic dysfunction and PVR increase in these patients, which can occur individually or collectively. Haemodynamic alterations regarding systemic venous congestion, RV-PA decoupling and diminished CI were not rare. Among those parameters, TAPSE and PVR were independently associated with 30-day mortality.

The placement of a central venous catheter is a routine procedure in septic patients in our ICU, which allows the measurements of CVP. CVP is mainly determined by cardiac function and venous return and normally is low. Earlier Surviving Sepsis Campaign guidelines recommended CVP 8–12 mmHg as a target of fluid management for sepsis patients in need of resuscitation [24]. Thus, some physicians are more likely to see the elevated CVP as a result of volume expansion rather than RV dysfunction. However, a high CVP can be defined as a value at which in most people the cardiac function curve is flat and not volume responsive [23]. A meta-analysis found that two thirds of the patients with a CVP < 8 mmHg were volume responders [25]. Antoine Vieillard-Baron et al. found that septic shock patients with CVP ≥ 8 mmHg and R/LVEDA ≥ 0.6 were less likely to responded to volume expansion [6]. Thus, in this study, we chose the cutoff value of CVP elevation as ≥ 8 mmHg. Even though CVP is an intraluminal pressure rather than a transmural pressure and its value can also be affected by the juxta-cardiac pressure, this study found that as much as 61% of the enrolled patients displayed at least one type of RV abnormality. Therefore, the elevated CVP can be seen as a warning sign of potential RV dysfunction, a timely RV function appraisal is warranted.

This patient cohort frequently displayed haemodynamic consequences. There is no normal CO, only one that is adequate or inadequate [26]. But CI below 2.5 was still seen as a criterion for cardiogenic shock [22]. We found 23% of the patients with CVP elevation had lower CI. RV-PA decoupling was either due to decreased RV systolic dysfunction or increased PVR or both, indicating RV cannot cope with its afterload. The TAPSE/PASP ratio has been proposed as a noninvasive indicator of RV-PA coupling [27]. We found that more than a third of the enrolled patients had a TAPSE/PASP ratio below 0.5 mm/mmHg. If CVP is high, the upper capillary pressure could be even higher. The systemic venous congestion indicates that the upstream organ perfusion is at a risk of being compromised. Interestingly, RV congestion based on R/LVEDA ≥ 0.6 and CVP ≥ 8 mmHg was found in 34% of the patients, but the systemic venous congestion based on IVC and hepatic vein Doppler was found only in 16% of the patients. Future research can be done to interpret the difference and connection between these two different assessment methods.

Among the measured RV-related parameters, we found only TAPSE and PVR were independently associated with 30-day mortality. TAPSE is the simplest and most reproducible parameter of RV systolic function [18]. There have been heterogeneous reports regarding TAPSE and prognosis in sepsis patients [28, 29]. Prior studies did not specifically test the relationship between TAPSE and prognosis, instead they tested the associations between RV systolic function and prognosis, using TAPSE as one of the RV systolic dysfunction parameters [9, 15, 30]. In a prior study, we found that using R/LVEDA, RV-PA coupling, and RV systolic indices including TAPSE, FAC and S’, could grade RV dysfunction [15]. Unfortunately, the RV grading score was complicated, which might limit the external validity of the results. Therefore, the combination of CVP and TAPSE might be a more simple way to assess RV function as well as to predict outcome of septic patients. In addition to chronic pulmonary hypertension, the prognostic relevance of PVR determined via echocardiography has been examined in subjects undergoing transcatheter aortic valve replacement [31]. The increase in RV afterload, resulting from acute respiratory distress syndrome, concomitant LV dysfunction, or positive pressure ventilation, is deemed as an important mechanism of RV dysfunction in sepsis [3, 4]. We found that echocardiography-based PVR can be obtained in a large proportion of septic patients, which could provide more simple and precise way of RV afterload estimation. Our results showed that increased PVR occurred in 14% of the patients. Unlike the LV, the RV has complex structural geometry, which render its echocardiographic appraisal more intricate [3]. This investigation aims to discern which echocardiographic indices bear the most substantial prognostic significance. Continuing studies are warranted to ascertain whether TAPSE and PVR may serve as effective therapeutic targets for the enhancement of septic patient prognosis.

### Limitations

Our study has several limitations. First, it is a single-centre retrospective study and our findings needs to be tested with data from other centres. Although it’s a retrospective study, the patients were prospectively screened and echocardiography was a routine examination in our ICU. Second, we did not collect data on RV strain due to both the constraints of image quality and the technical limitations inherent to portable echocardiographic devices. The prognostic importance of RV longitudinal strain has been evaluated in cohorts without sepsis and in paediatric populations with sepsis [32–34]. Future research can be done to contrast the prognostic value of RV strain against that of standard echocardiographic measures in septic patients with elevated CVP. Third, some measurements for the collected RV parameters were unavailable, which might underestimate the proportion of RV abnormality. Despite these limitations, we hope this study might shed a light on the RV assessment based on CVP and echocardiographic parameters.

## Conclusions

Echocardiographic findings demonstrated a high prevalence of RV-related abnormalities (RV enlargement, RV systolic dysfunction and PVR increase) in septic patients with CVP ≥ 8 mmHg. Among those echocardiographic parameters, TAPSE and PVR were independently associated with 30-day mortality in these patients.

### Supplementary Information

**Supplementary Material 1.**

## Data Availability

All datasets used and analyzed during the current study are available from the corresponding author on reasonable request.

## Abbreviations

CVP: Central venous pressure
RV: Right ventricle
R/LVEDA: Right and left ventricular end-diastolic area ratio
PVR: Pulmonary vascular resistance
CI: Cardiac index
ICU: Intensive care unit
TAPSE: Tricuspid annular plane systolic excursion
FAC: Fractional area change
S’: Tissue Doppler peak velocity of tricuspid annulus
R/LVEDA: Right and left ventricular diastolic area ratio
MAP: Mean arterial pressure
LVOT: Left ventricular outflow tract
TR: Tricuspid regurgitation
VTI: Velocity–time integral
IVCD: Inferior vena cava diameter
PA: Pulmonary artery
PASP: Pulmonary arterial systolic pressure
HV: Hepatic vein
HR: Heart rate
NE: Norepinephrine
APACHE: Acute physiology and chronic health evaluation
SOFA: Sequential organ failure assessment
PEEP: Positive end-expiratory pressure
Pplat: Plateau pressure
