# Supplementary material for: Echocardiographic features of right ventricle in septic patients with elevated central venous pressure
Source: BMC Anesthesiol. 2024 Apr 4;24:128. doi: 10.1186/s12871-024-02515-8 (PMC10993580; doi:10.1186/s12871-024-02515-8)
Supplement: Supplementary file 1 — Supplementary Material 1. [file 12871_2024_2515_MOESM1_ESM.docx]

Supplemental Table 1. Echocardiographic parameters

| Categories | Findings |
| --- | --- |
| LVEF(%) | 59 (48, 66) |
| MAPSE(mm) | 13.4 (10.8, 15.8) |
| LVOT-VTI | 16.6 (13.3, 19.4) |
| E(cm/s) | 66(53, 84) |
| e’(m/s) | 8.0 (6.2, 9.8) |
| IVCD (cm) | 1.9 (1.6, 2.2) |
| HV S< D (n, %) | 44 (18) |
| TR(m/s) | 2.3 (2.1, 2.6) |
| RVOT-VTI | 11.2 (9.1, 14.1) |

LVEF: left ventricular ejection fraction; MAPSE: mitral annular plane systolic excursion; LVOT: left ventricular outflow tract; VTI: velocity-time integral; E: early transmitral inflow velocity; e’: early diastolic tissue Doppler velocity of mitral annulus; IVCD: inferior vena cava diameter; HV: hepatic vein; TR: tricuspid regurgitation; RVOT: right ventricular outflow tract.

Supplemental Table 2. Factors associated with 30-day mortality

|  | HR | 95%CI | *p* |
| --- | --- | --- | --- |
| **Univariable analysis** |  |  |  |
| Age | 1.021 | 1.005-1.037 | 0.012 |
| Sex | 0.757 | 0.434-1.321 | 0.328 |
| SOFA | 1.328 | 1.209-1.459 | <0.001 |
| APACHEⅡ | 1.132 | 1.097-1.169 | <0.001 |
| Lung infection | 3.235 | 1.892-5.531 | <0.001 |
| PEEP | 1.172 | 1.058-1.298 | 0.002 |
| Pplat | 1.127 | 1.078-1.177 | <0.001 |
| Norepinephrine | 2.109 | 1.639-2.713 | <0.001 |
| R/LVEDA | 1.328 | 1.128-1.564 | 0.001 |
| TAPSE | 0.303 | 0.173-0.528 | <0.001 |
| S’ | 0.896 | 0.819-0.981 | 0.018 |
| FAC | 0.962 | 0.942-0.983 | <0.001 |
| RVOT-FS | 0.9921 | 0.971-1.013 | 0.421 |
| PVR | 1.731 | 1.306-2.294 | <0.001 |

SOFA: sequential organ failure assessment; APACHE: acute physiology and chronic health evaluation; PEEP: positive end-expiratory pressure; Pplat: plateau pressure; R/LVEDA: right and left ventricular end-diastolic area ratio; TAPSE: tricuspid annular plane systolic excursion; S’: tissue Doppler peak velocity of tricuspid annulus; FAC: fractional area change; RVOT-FS: right ventricular outflow tract fractional shortening; PVR: pulmonary vascular resistance.
